# Fixed-target serial oscillation crystallography at room temperature

**DOI:** 10.1107/S2052252519001453

**Published:** 2019-02-23

**Authors:** Jennifer L. Wierman, Olivier Paré-Labrosse, Antoine Sarracini, Jessica E. Besaw, Michael J. Cook, Saeed Oghbaey, Hazem Daoud, Pedram Mehrabi, Irina Kriksunov, Anling Kuo, David J. Schuller, Scott Smith, Oliver P. Ernst, Doletha M. E. Szebenyi, Sol M. Gruner, R. J. Dwayne Miller, Aaron D. Finke

**Affiliations:** a MacCHESS, Cornell University, Ithaca, NY 14853, USA; b Departments of Chemistry and Physics, University of Toronto, Toronto, ON Canada; c Max Planck Institute for the Structure and Dynamics of Matter, Hamburg, Germany; d Departments of Biochemistry and Molecular Genetics, University of Toronto, Toronto, Ontario, Canada; e Department of Physics, Cornell University, Ithaca, NY 14853, USA; f Kavli Institute for Nanoscale Science, Cornell University, Ithaca, NY 14853, USA

**Keywords:** fixed-target serial oscillation, serial crystallography, oscillations, structural biology, radiation damage, structure determination, X-ray crystallography, storage rings

## Abstract

A fixed-target approach to high-throughput room-temperature serial synchrotron crystallography with oscillation is described. Patterned silicon chips with microwells provide high crystal-loading density with an extremely high hit rate. The microfocus, undulator-fed beamline at CHESS, which has compound refractive optics and a fast-framing detector, was built and optimized for this experiment. The high-throughput oscillation method described here collects 1–5° of data per crystal at room temperature with fast (10° s^−1^) oscillation rates and translation times, giving a crystal-data collection rate of 2.5 Hz. Partial datasets collected by the oscillation method at a storage-ring source provide more complete data per crystal than still images, dramatically lowering the total number of crystals needed for a complete dataset suitable for structure solution and refinement – up to two orders of magnitude fewer being required. Thus, this method is particularly well suited to instances where crystal quantities are low. It is demonstrated, through comparison of first and last oscillation images of two systems, that dose and the effects of radiation damage can be minimized through fast rotation and low angular sweeps for each crystal.

## Abstract 2

The combination of oscillation data collection with fixed-target microchips for regular crystal dispersion is an efficient method for collecting serial crystallography data at synchrotrons. Background scatter from noncrystal substrates is especially minimized.

## 1. Introduction   

X-ray crystallography has been the predominant technique for structure elucidation of biomacromolecules for the past 30 years. The success of an experiment hinges on whether the specimen under investigation can be crystallized with sufficient size and quality (Holton & Frankel, 2010 ▸). Two major challenges facing structural biologists in the next decade will be the analysis of large, complex systems and room-temperature analysis of protein folding (Fraser *et al.*, 2011 ▸; Grimes *et al.*, 2018 ▸). While complementary techniques such as NMR, solution X-ray scattering and especially cryo-EM have taken great strides to address these, X-ray crystallography remains a stalwart method in the structural biologist’s toolbox. But that does not mean crystallography is solely a routine tool; many new and exciting advancements have been developed to address the changing demands of structural biologists and the development of high-brilliance coherent light sources such as X-ray free-electron lasers (XFELs) and ultra-low-emittance storage-ring (SR) light sources.

One such technique is serial crystallography (SX), in which partial datasets taken from many individual crystals are merged together. This method has found great utility, particularly for the diffraction of microcrystals (Schlichting, 2015 ▸; Gruner & Lattman, 2015 ▸; Martin-Garcia *et al.*, 2016 ▸). The brand of SX developed for crystallography at XFELs, serial femtosecond crystallography (SFX), operates on the ‘diffraction before destruction’ principle – collect the diffraction pattern from a crystal before the crystal itself is destroyed by the Coulomb explosion resulting from the X-ray pulse (Chapman *et al.*, 2011 ▸). This method has produced a large number of high-profile structures (Kang *et al.*, 2015 ▸; Liu *et al.*, 2013 ▸; Redecke *et al.*, 2013 ▸; Stauch & Cherezov, 2018 ▸; Aoyama *et al.*, 2009 ▸; Suga *et al.*, 2015 ▸) and, excitingly, has been used to conduct time-resolved dynamics experiments (Barends *et al.*, 2015 ▸; Nango *et al.*, 2016 ▸; Pande *et al.*, 2016 ▸; Nogly *et al.*, 2018 ▸). The extremely short pulse duration in SFX means that the diffraction pattern recorded from the XFEL pulse is a ‘still’ image, in which crystal motion during irradiation is negligible. Only partial reflections are recorded, so in order to capture the full intensity of each Bragg peak, multiple data frames must be scaled and merged. Since only a single frame, containing partial reflections, is recorded from each crystal before it is destroyed and since high redundancy is necessary to obtain accurate intensities, the number of crystals required to generate a complete dataset is very high. Additionally, when the crystals are delivered to the beam randomly with no control over the orientation a further increase in the number of crystals is necessary to sample reciprocal space properly. A huge effort to optimize integration, scaling and merging routines for still diffraction data has lowered the total amount of data needed in SFX (Lyubimov *et al.*, 2016 ▸; Sauter, 2015 ▸; Uervirojnang­koorn *et al.*, 2015 ▸; White, 2014 ▸; White *et al.*, 2016 ▸), but multiple thousands of crystals are still typically required.

Serial synchrotron crystallography (SSX), or serial millisecond crystallography, is complementary to SFX and is performed at SR light sources. While even the brightest SR light sources are currently orders of magnitude less brilliant than XFELs, there are still a number of advantages to using SR radiation. First is the current high availability of SR beamlines for crystallography. Second is the fact that SR radiation has finer tuning of beam properties such as bandwidth (Meents *et al.*, 2017 ▸), energy and flux, as well as better beam stability and reliability (at least currently). Third is the possibility of performing oscillation. Oscillation crystallography, which is the standard method for protein crystallography, samples a wide area of reciprocal space per data frame by rotating the sample while exposing it to X-rays, resulting in better reflection profiles (Arndt & Wonacott, 1977 ▸). X-ray exposure with oscillation allows for more of the reciprocal space to be sampled per frame, compared with a still image at an equivalent dose. Oscillation is not possible when using XFELs as the femtosecond X-ray pulse time renders negligible any macroscopic crystal motion during exposure, although goniometer-based approaches have proven useful for collecting a series of stills that can be merged into a pseudo-oscillation dataset, when a sufficiently large crystal mounted on a fixed target is available (Cohen *et al.*, 2014 ▸; Hirata *et al.*, 2014 ▸).

Because SSX requires diffraction data from a large number of crystals, delivery of the crystal samples to the X-ray beam presents a logistical challenge. In addition to the small size of the crystals that need to be delivered to the X-ray beam, one must also consider the X-ray scattering produced by the delivery vector, which is parasitic to the desired crystalline diffraction and contributes to noise. Broadly, there are two methods for serial sample delivery: moving-target methods, where slurries of crystal specimens dispersed in a matrix such as a solution or lipidic cubic phase (LCP) are sprayed as a narrow jet or series of droplets across the beam path with continuous data collection; and fixed-target methods, where crystals are suspended on a substrate, which is then translated across the beam. Both are useful methods, each with its own advantages. Jets allow for the introduction of crystals into the beam in their own growth or stabilization media, including LCP (Botha *et al.*, 2015 ▸; Martin-Garcia *et al.*, 2017 ▸; Nogly *et al.*, 2015 ▸; Stellato *et al.*, 2014 ▸). However, jets require more crystals as the hit rate of crystals that provide diffraction data can be low. Additionally, parasitic scattering from the jet media can be severe, especially with LCP.

Fixed-target methods in SSX allow for higher crystal hit rates and the potential for minimizing background scatter (Zarrine-Afsar *et al.*, 2012 ▸; Axford *et al.*, 2015 ▸; Coquelle *et al.*, 2015 ▸; Oghbaey *et al.*, 2016 ▸; Owen *et al.*, 2017 ▸; Melnikov *et al.*, 2018 ▸). Several different fixed-target approaches have been developed, including sample grids (Cherezov *et al.*, 2009 ▸; Murray *et al.*, 2015 ▸; Roedig *et al.*, 2015 ▸; Zander *et al.*, 2015 ▸), microfluidic devices (Dhouib *et al.*, 2009 ▸; Gicquel *et al.*, 2018 ▸; Heymann *et al.*, 2014 ▸; Perry *et al.*, 2013 ▸) and even *in vivo* (Boudes *et al.*, 2016 ▸) and *in situ* (Oghbaey *et al.*, 2016 ▸; Huang *et al.*, 2016 ▸) methods. In contrast to moving-target methods, where the crystals are discarded after collection, fixed-target methods allow samples to be re-exposed to the beam – a process which we have recently exploited (Schulz *et al.*, 2018 ▸). Some of these approaches minimize the amount of materials within the X-ray exposure path by using low-scattering thin materials, such as graphene, thus minimizing the background-scattering contributions from sources other than the crystal itself and improving the signal-to-noise ratio (SNR) (Sui *et al.*, 2016 ▸; Wierman *et al.*, 2013 ▸).

We have developed a fixed-target method for serial crystallography in which silicon chips are patterned with micrometre-sized wells in a regular grid (Mueller *et al.*, 2015 ▸; Owen *et al.*, 2017 ▸). Crystals are suspended in the wells and kept hydrated with minimal solution, then sealed with ultra-thin membranes. This reduces background scatter from the substrate and the solution, as well as minimizing the chance for multiple crystals to inhabit a single well. The hit rate for crystals suspended in precisely defined locations is high, and we have developed programs for fast grid rastering. Locating crystals on the chips prior to exposure further decreases the amount of time needed to collect data (Oghbaey *et al.*, 2016 ▸).

Here, we describe a high-throughput approach to serial oscillation crystallography (Hasegawa *et al.*, 2017 ▸). Using the silicon microchip technology with fast translation that has proven to be useful for serial collection of still data from crystals at XFELs and synchrotrons, we have found that adding oscillation to crystal data collection dramatically decreases the number of crystals needed to generate a complete dataset, compared with still data collection. By optimizing oscillation rate and dose, the effects of radiation damage can be minimized while the amount of data collected is maximized.

## 2. Methods and materials   

### 2.1. Protein crystallization   

Fluoro­acetate dehalogenase (FAcD) was prepared and purified as described previously (Chan *et al.*, 2011 ▸; Kim *et al.*, 2017 ▸; Schulz *et al.*, 2018 ▸). Large FAcD crystals were obtained using the hanging-drop method with 0.5 m*M* FAcD and a mother liquor of 16–20% PEG 3350, 100 m*M* Tris–HCl pH 8.5, and 200 m*M* CaCl_2_. We prepared a microseed stock from the large FAcD crystals using a Hampton Research seed bead kit (HR2-320); batch crystallization was carried out using a 1:1 ratio of the microseed stock to 0.5 m*M* FAcD solution. Crystals were 20 × 20 × 20 µm.

Hen-egg-white lysozyme was purchased from Sigma and 50 mg ml^−1^ of lysozyme solution (in deionized H_2_O) was used for crystallization. A mixture of 1 ml of lysozyme sample and 3 ml of precipitant [20%(*w*/*v*) NaCl, 6% PEG 6 K, 0.5 *M* NaOAc pH 4.0] was prepared and stored at 4°C. Crystals appeared after 24 h (size = 10 × 10 × 10 µm). The solution in the tube was replaced by a storage solution [8%(*w*/*v*) NaCl, 0.1 *M* NaOAc pH 4.0]. The crystals grew slightly bigger (size = 40 × 40 × 40 µm) after 24 h in the storage solution. All tubes with lysozyme crystals were kept at 4°C.

Sperm whale myoglobin (SWMb) was purified as described previously (Springer & Sligar, 1987 ▸; Mueller *et al.*, 2015 ▸) with some modifications. Expression and purification of SWMb is described in detail in Section S1 of the Supporting information. To ensure the SWMb crystals were grown in the CO-bound form (CO-SWMb) all crystallization buffers were saturated with CO gas. The CO-SWMb crystals were grown in a 3 ml Monoject blood collection tube (Covidien, Mansfield, USA) that was prefilled with 1 atm (1 atm = 101325 Pa) of CO gas. Seeding was used to generate large quantities of small crystals. A micro-vial homogenizer (BP-7005-000) from Wilmad-LabGlass SP Scienceware (Vineland, NJ, USA) was used to break up the crystals into very small sizes. The CO-SWMb crystals were homogenized in 10 m*M* Tris–HCl pH 9.0 and 3.2 *M* NH_4_)_2_SO_4_ and washed two to three times with the same CO-saturated solution. The washed crystals were collected by centrifugation. The seeds were diluted 1:200 under the same buffer conditions. Each vial was injected with 180–200 µl of crystallization solution containing 12–13 mg ml^−1^ protein in 10 m*M* Tris–HCl pH 9.0 and 2.5–2.6 *M* (NH_4_)_2_SO_4_ pre-saturated with CO. To promote nucleation of large quantities of small crystals, 10 µl of prepared seed stock was injected into the tube. Suitable sizes of final crystals were obtained by adding ∼2 ml of 10 m*M* Tris–HCl pH 9.0 and 3.2 m*M* NH_4_)_2_SO_4_ pre-saturated with CO to each tube after crystals appeared. Crystals were 30 × 30 × 30 µm in size.

### 2.2. Beamline setup   

**Figure 1:**
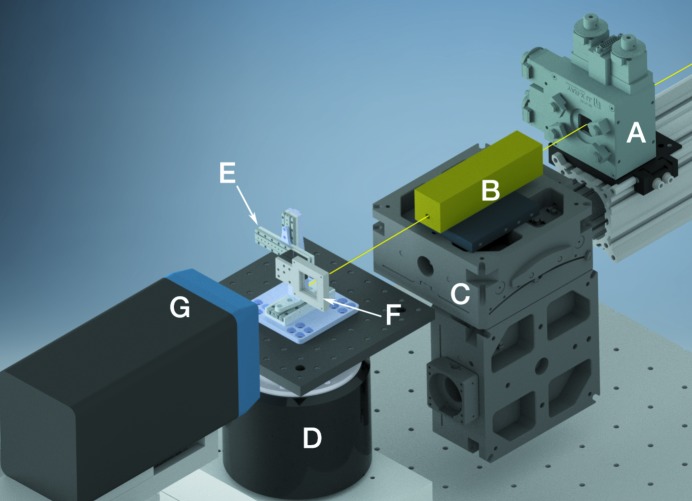
Schematic of major beamline components. Shown is the arrangement with oscillation about the *Z* axis. The beam path is shown in yellow. A, beam-defining slit; B, CRL box with aperture; C, CRL positioning motors; D, air-bearing goniometer; E, three-axis piezo translation stage; F, chip holder; G, EIGER detector.

Data were collected at beamline G3 of CHESS. G3 is an undulator-fed beamline with a dual W/B_4_C multilayer monochromator and a flat multilayer focusing mirror at 1.5% bandwidth. This produces the high flux necessary for narrow beams desired for collecting diffraction data from small crystals. Fig. 1 ▸ shows the beamline setup.

The bandwidth of this setup is higher than a more typical protein crystallography beamline with a silicon-based monochromator. This was chosen to increase the number of photons entering the sample. Serial Laue crystallography using ‘pink beams’ of 4–5% bandwidth has been demonstrated to reduce the number of crystal samples needed (Meents *et al.*, 2017 ▸). Data collected at 1.5% bandwidth is sufficiently monochromatic to be processed with standard data-reduction software, offering a good compromise between Laue and monochromatic methods.

#### 2.2.1. Microbeam focusing with compound refractive lenses   

Smaller crystals require narrow beams and high flux density to optimize diffraction. At the G3 beamline of CHESS, we installed a stack of 31 beryllium compound refractive lenses (CRLs) (RXOptics), which focus 2.0 × 10^11^ photons s^−1^ at 10.2 keV (1.216 Å) into a 7 × 9 µm(*V* × *H*) (FWHM of Gaussian profile) focal waist 250 mm from the middle of the stack (Snigirev *et al.*, 1996 ▸, 1998 ▸).

Additionally, the X-ray beam upstream of the CRL stack was collimated via a series of slits (beam-defining and guard) to reduce scatter and ensure optimal illumination upon the entrance to the CRL stack. Matching the upstream beamsize closely to the diameter of the CRL entrance (350 µm) proved optimal. A 200 µm guard aperture downstream from the CRL stack removed parasitic scatter from helium, air and the stack itself.

#### 2.2.2. Background reduction   

At every point in the experiment, minimizing parasitic scatter from any material in the beampath is integral to improving the SNR of crystal diffraction. We used thin window materials, helium flight paths and sample-chamber environments wherever possible. Kapton windows (8 µm thickness) separated the helium flight path containing the ion chambers and the CRL stack from upstream air. A large Mo beamstop (700 µm) was placed downstream from the sample position to eliminate scatter generated by the focused beam by acting on the atmosphere within the sample chamber up to the detector. Finally, we installed a positive-helium-pressure sample chamber (a translucent polyethyl­ene bag) from the end of the aperture housing to the detector. It also enclosed the sample position, the inline camera and the downstream beamstop. This simple enclosure reduced the background scatter by a factor of two or more.

#### 2.2.3. Translation stages and goniometer   

Regular positioning of wells on chips ensures high hit rates. An air-bearing goniometer (Aerotech), mounted with the rotation axis perpendicular to the X-ray beam waist and controlled by a servo controller (Parker Motion), was used for sample oscillation. The oscillation velocity was 10° s^−1^; current hardware constraints inhibited faster oscillation. The axis of oscillation can either be along the *X* or the *Z* axis; Fig. 1 ▸ shows a beamline setup with oscillation about the *Z* axis. Both orientations are suitable for collecting data, and each has its own strengths. A setup with oscillation about the *X* axis is better suited for ‘typical’ protein crystallography beamline setups that have oscillation about the *X* axis but the torque applied by the translation stages can lead to hardware fatigue and increased sphere of confusion. Oscillation about the *Z* axis is nonstandard but alleviates these concerns. We have collected data using both orientations without issue and the diffraction data in this study was taken with oscillation about the *Z* axis as shown in Fig. 1 ▸.

We use fast piezo stages of a design identical to that described by Sherrell *et al.* (2015 ▸) to translate the chip in the holder to fixed positions. We used two SLC-1750 ultrafast positioners (SmarAct) for *X* and *Z* translations and a single SLC-2450 positioner (SmarAct) for translation in the *Y* direction (parallel to the beam); the latter was useful for determining the beam-waist position. These positioners use piezoelectric motors that are driven with an SDC controller (SmarAct). For higher level control, we used a Geobrick LV-IMS-II (Delta Tau) for simultaneous and synchronizing motions. Positioner control and alignment was carried out using the *PEwin32* *Pro 2* software (Delta Tau), using a two-coordinate system in the *xz* plane orthogonal to the beam axis, defining the top-left, top-right and bottom-right wells to determine the precise chip alignment. The GeoBrick controller updates positions and velocities for all three axes every 450 µs and corrects the desired position with a built-in closed-loop amplifier proportional-integral-derivative controller. Wells can be visualized in one of two ways: with an in-line camera attached to a microscope for optical visualization or with X-rays and active framing of the detector to minimize silicon scattering, which occurs at defined positions around 1.6 Å. The former is cruder but faster, the latter slower but more accurate. Chip alignment using X-rays takes less than three minutes.

#### 2.2.4. Hardware synchronization   

A critical component of this setup is timing oscillation to coincide with detector-exposure framing and subsequent pausing of data collection, while the translation stages move the next sample well into the beam. Between collecting data frames while rotating the sample, translating to a new sample and repeating data collection on the new sample for thousands of samples, all moving motors must be correctly synchronized for the desired oscillation and exposure. Synchronization was maintained through precise timing from the servo controller of the air-bearing goniometer (Aerotech) combined with a delay generator to trigger detector framing.

The servo motor controller for the air-bearing is connected to a delay generator (Stanford), and to the translation-stage controller via a voltage stepper that converts the 5 V TTL (transistor–transistor logic) signal from the servo to 18 V for the translation-stage controller. At the beginning of the oscillation motion, a 5 V TTL signal is sent from the servo controller to the delay generator and to a fast shutter (Uniblitz), which is set to open for positive TTL signals. The delay generator forces a delay of 69 ms to account for acceleration of the goniometer and the shutter opening (12 ms). The delay generator then sends out 5 V TTL pulses of a defined length, equal to 0.2° oscillation per pulse (20 ms at 10° s^−1^ oscillation rate), to the detector, with a delay of 10 µs between pulses. The number of pulses is dependent on the total oscillation and the oscillation angle per frame: for 0.2° oscillation per frame 5 pulses are sent for 1° oscillation, 15 pulses for 3° *etc.*

A correction has to be made because goniometer oscillation accelerates at a finite rate. All oscillations were performed with an extra 0.5° at the beginning and end of oscillation; thus, a 1° measured oscillation moved a total of 2° *etc.*, ensuring reliably repetitive movement. Upon completion of the oscillation, the servo sends a −5 V TTL signal out, closing the shutter. The Geobrick is triggered to move the translation stage the distance of one well. Translation is fast (<10 ms). To simplify data reduction, data were collected during rotation in only one direction, *i.e.* the 5 V TTL signal is only sent out in one oscillation direction. Thus, the goniometer moved back to its initial state before resuming data collection while the translation stage was moving the chip to the next well. Collection of oscillation data in this manner is currently about an order of magnitude slower than ‘still’ data collection with the same translation hardware (2.5 Hz for oscillation data collection versus 30+ Hz for still). A video of the oscillation and translation, with the oscillation axis in the *X* direction, is available in the Supporting information.

#### 2.2.5. Detector   

In order for data collection to be performed in a reasonable amount of time, a fast-framing detector is required. We used an EIGER 1M detector (Dectris) for data collection. The EIGER enables fast, shutterless data collection with negligible ‘dead’ time between frames, and features a small pixel size and single photon counting. As described above, detector framing is controlled by pulses sent by the delay generator. Collecting data frames in so-called ‘fine phi-sliced’ mode, as done here, has been shown to improve data quality in shutterless data collection (Mueller *et al.*, 2012 ▸; Casanas *et al.*, 2016 ▸). Thus, framing was done in increments of 0.2° oscillation per frame. At 10° s^−1^ oscillation rate, this corresponds to a frame rate of 50 Hz. The dead time in between frames is 10 µs, giving a data loss of 0.05%.

### 2.3. Fixed-target silicon chips   

**Figure 2:**
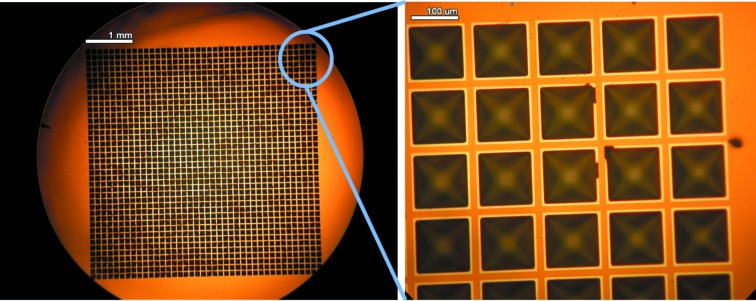
Optical microscopy image of a fixed-target chip containing one grid of 40 × 40 microwells.

Chips were prepared as described previously (Mueller *et al.*, 2015 ▸; Oghbaey *et al.*, 2016 ▸; Sherrell *et al.*, 2015 ▸). Each chip consists of 1, 4 or 9 grids of 40 × 40 well features, for a total of 1600, 6400 or 14 400 wells, respectively. Each well feature has an opening size of 110 ± 5 µm on the top and 20 ± 5 µm at the bottom (Fig. 2 ▸). The wells were 150 µm deep. The size of the well can be modified by the length of the etch time, anywhere from 5 to 100 µm. The pit shape of the feature is ideal for trapping a crystal and removing the excess liquid in it. The exact positioning of the wells in an array, with submicrometre accuracy, enables very precise access to individual crystals with no further need for rastering or pre-scanning on the beamline.

The ability to precisely position microcrystal samples and translate to them quickly increases the sampling rate; the possible addition of inline mapping (Oghbaey *et al.*, 2016 ▸) will increase the already high hit rate. An added benefit of the design of the chip is the isolation of crystal samples within individual wells; this eliminates radical diffusion from one sample to the next, ensuring radiation-free ‘fresh’ samples with every new exposure. Perhaps most importantly, the fixed-target approach minimizes the sources of background scatter, thus maximizing the SNR of even weak diffraction from microcrystals.

### 2.4. Sample loading   

Sample loading was performed at a temperature of 20°C and a humidity of 60% in order to prevent salt-crystal formation. To control the humidity, all loading was done within a homemade, mobile, acrylic glove box equipped with a humidifier. Depending on crystal density of the sample, between 200 and 500 µl of crystal suspension was transferred using a pipette to the top of the chip. A gentle suction was applied from below the chip in order to pull the crystals into the features of the chip. If crystals are robust, a vacuum can be applied, but for sensitive crystals simply wicking moisture through the wells with a paper towel is effective. For the crystals in this study, a vacuum was applied. The loaded chip was sandwiched into a custom holder and quickly covered, front and back, with 3 µm Mylar film in order to prevent crystals from drying out during room-temperature data collection. The chip structure allows a large number of microcrystals to be loaded using turbulent fluid flow, which introduces random orientation of crystals to ensure sufficient sampling of reciprocal space. A typical crystal was 30–50 µm in diameter.

### 2.5. Data collection   

The sample holder containing the chip was placed on the translation stage and the helium bag chamber closed. The chip was oriented with the wells pointing toward the detector so the beam path was limited only to the well-hole size. After chip alignment, as described in Section 2.2.3, data collection was initiated. All data collection was performed at room temperature. For 6400 wells and 1° oscillation, data collection was complete in about 40 min.

### 2.6. Data processing   

Data processing occurred broadly in three steps: (1) an initial screening step to determine which datasets have indexable Bragg peaks, which are then merged to determine the unit cell and overall crystal symmetry; (2) reprocessing with the correctly determined space group and crystal system; and (3) a filtering and optimization of the merging step. The data-processing strategy was adapted from the one described on the XDSWiki (https://strucbio.biologie.uni-konstanz.de/xdswiki/index.php/SSX-PepT_Se).

Individual datasets were processed with *XDS* (Kabsch, 2010 ▸) using a bash script for automation (Section S5 in the Supporting information). A full *XDS* run takes a few seconds on a modern multicore processor if parallel execution is run (xds_par). If there are insufficient spots found for indexing, if indexing or refinement fails, or if integration fails, *XDS* ends without producing a list of Miller indices, intensities and sigmas (the XDS_ASCII.HKL file). In this way, each *XDS* run is its own triage for a dataset. The IDXREF step of *XDS* is a critical filter for data quality as it defaults to terminate the program if less than 50% of reflections are indexable to a single unit cell. The maximum error and minimum fraction of indexed spots needed can be relaxed if the defaults are too restrictive but in our experience they do not need to be changed.

Datasets were initially processed in space group *P*1. Because each dataset contained, at most, a few degrees of oscillation data, there was usually not enough data to find information about the crystal system or space group symmetry – only the reduced cell. However, the reduced cell was usually sufficient to filter out datasets that were significant outliers (*e.g.* salts).

Datasets were merged with *XSCALE*. The initial reference dataset for scaling was chosen arbitrarily. The initial merging step was done in *P*1 with the average reduced unit cell. The crystal system and point group were then determined with *POINTLESS* (Evans, 2011 ▸), part of the *CCP*4 suite (Winn *et al.*, 2011 ▸). In cases where there was indexing ambiguity, the highest symmetry space group with a significant probability was chosen. The individual datasets were then reprocessed with the correct unit cell and space group in *XDS*. This time, only the INTEGRATE and CORRECT steps needed to be run. Correction factors were not applied for individual datasets; this was done later during the final scaling steps. *XSCALE* was then rerun. Data filtering and optimization were done with the program *XSCALE_ISOCLUSTER*, using the CC clustering method (Brehm & Diederichs, 2014 ▸; Diederichs, 2017 ▸). We filtered outlying datasets based on the strength (SNR) and distance away from the center of the cluster. We did not require multiple clusters for any of our datasets. Datasets with a calculated strength × cos(θ) < 0.6, where θ is the angle from the center of the cluster, were removed. The filtered datasets were then rescaled together with *XSCALE*, with the dataset with the highest strength × cos(θ) chosen as the reference dataset. Corrections for decay, modulation and absorption were applied only in the final step. Data-resolution cutoffs were set to the CC_1/2_ cutoff given by *XSCALE*.

### 2.7. Structure solution and refinement   

The structure was solved by molecular replacement using *PHASER* (McCoy *et al.*, 2007 ▸) as a component of *PHENIX* (Adams *et al.*, 2010 ▸), with starting models from the Protein Data Bank: FAcD (PDB entry 6fsx; Schulz *et al.*, 2018 ▸); lysozyme (PDB entry 1dpx; Weiss *et al.*, 2000 ▸) and native SWMb without CO (PDB entry 1vxa; Yang & Phillips, 1996 ▸). Refinement was completed with the *phenix.refine* routine of *PHENIX*. TLS domains were identified using the *TLSMD* server, and the TLS domains were used in the final stages of refinement (Painter & Merritt, 2006 ▸).

## 3. Results and discussion   

**Figure 3:**
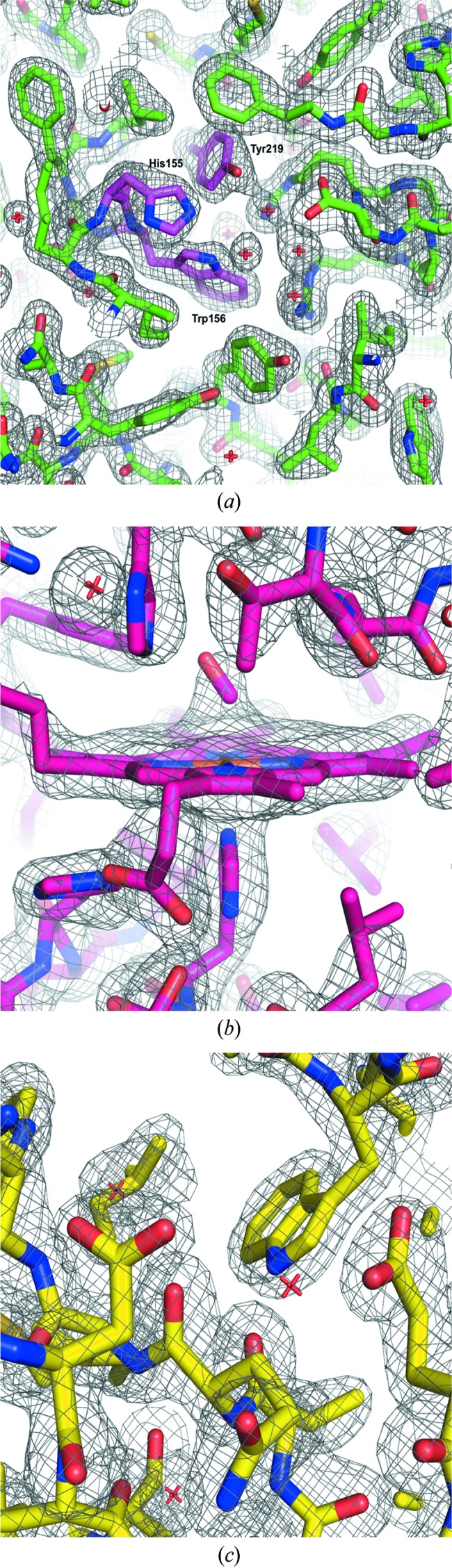
Composite OMIT maps (2*mF*_o_−*DF*_c_) of selected structures. Maps were calculated using *PHENIX* (Adams *et al.*, 2010 ▸). Maps are contoured at 1σ. (*a*) FAcD from Chip 1, pictured here around the active site with the residues responsible for catalysis labeled and shown in magenta. (*b*) Carb­oxy­myoglobin, pictured here around the heme moiety. (*c*) Lysozyme, around the active site.

We tested three different protein crystals for this study: FAcD, lysozyme and CO-SWMb. The results of the data collection and refinement are shown in Table 1 ▸. Fig. 3 ▸ shows electron-density maps of the active sites of FAcD (from Chip 1), CO-SWMb and lysozyme.

What is most striking about the combination of a fixed-target approach with oscillation is crystal economy: compared with collection of still images, the oscillation method requires far fewer crystals in order to generate a complete dataset. For example, in a previous work using the chip setup with CO-SWMb crystals (Mueller *et al.*, 2015 ▸), still data from 1776 crystals were required in order to generate a complete dataset; in our work, with 5° oscillations per crystal, only 138 crystals were needed. This is likely to be a combination of both oscillation and the relatively large bandwidth of the X-rays. We are currently assessing the benefits and drawbacks of higher-bandwidth X-rays on crystallographic experiments.

Recently we reported a time-dependent crystallographic study on the ligand binding of FAcD using our chip system; the experiment necessitated that diffraction data were collected as stills (Schulz *et al.*, 2018 ▸). A complete FAcD dataset required the collection of data from >10 000 crystals, with at least 3318 images used in the final refinement; in this study, with 1° oscillation, we collected 2626 datasets and only 494 were used in the final refinement. Economy of crystal usage is an aspect that is often overlooked in discussions of serial crystallography, to its detriment. High-value targets may produce more microcrystals than large-sized ones but the number is still finite and likely to be much smaller than with more common macromolecules. Methods to minimize the number of crystals needed will help ensure successful application of SSX. Oscillation, while slower to collect data from each crystal, generates far more data per crystal than still collection, and more than makes up for the additional time in terms of data quality and scale. For 6400 wells using 1° oscillation, well collection is carried out in 40 min, and with on-line scaling and filtering, one can obtain a complete dataset and solved structure in under one hour. With automated, ‘on-the-fly’ data reduction and processing combined with high-throughput oscillation, the total amount of data needed for a complete dataset and the amount of time spent collecting data can be minimized.

The total amount of data that can be extracted from a single crystal in oscillation mode is limited by three factors: the SNR of the diffracting crystals, the maximum oscillation range, and radiation damage. Minimizing sources of background scatter to maximize SNR is critical for collecting data from weakly diffracting crystals. We tested several different window materials and found that 3 µm Mylar gave a good SNR while ensuring a watertight seal around the chip. Other materials, such as Kapton or cyclic olefin copolymer, were less suitable (Bish *et al.*, 2014 ▸; Broecker *et al.*, 2016 ▸). Removing as much as possible of the mother liquor from the crystals without drying them out was also a critical step. Wicking the solution through the well with a dry paper towel proved the gentlest and most effective means of liquid removal, and gave us better control over how much liquid was retained on the chip. Scattering from the crystalline silicon substrate appears as regular diffraction patterns that can be masked out. Even with non-ideal alignment and the presence of the Si diffraction, which consists of six large peaks at around 1.6 Å, the peaks are easily masked during data processing. This turns out to be a major advantage for minimizing background scatter, as the only sources of diffuse scatter that have an impact on the background are the Mylar windows, helium scatter and any liquid that may still surround the crystal. Lastly, collecting data at room temperature in an enclosed system allowed for data collection in a helium atmosphere, further reducing background scatter.

The maximum oscillation range – that is, the maximum rotation possible before substrate scatter or physical limitations prevent collection of more data – was acceptable in our system for two reasons. First, the setup allows for up to 90° oscillation without collisions. Second, the beam size used in this study (9 µm horizontal) is about half that of the well size (20 µm), allowing for a wide tilt from the perpendicular before the beam began to strike the edge of the well. We were able to perform oscillations of up to 15° with proper alignment.

The oscillation wedge angle that is collected on each crystal has a dramatic effect on the total number of crystals needed to generate a complete dataset. To demonstrate this, data were collected on chips containing FAcD with two different oscillation angles: 1° (Chip 1) and 3° (Chip 2). The total number of crystals needed for final refinement of Chip 2 was about half that needed for Chip 2 (Table 1 ▸). Both chips gave good quality datasets suitable for structure solution and refinement, with no significant differences between refined structures. Since different crystals and different chips were used for these datasets, we performed a further test by independently processing the first 1° data and last 1° data from crystals of Chip 2. It was found that 394 and 428 datasets were required to give a complete dataset from the first 1° and last 1° of crystals from Chip 2, respectively (Supporting information). Both datasets gave similar, if slightly poorer, processing statistics than the full 3° data. Of the 294 crystals used for the refinement of the 3° dataset, 164 (56%) and 181 (62%) of those were used in the refinement of the first 1° and last 1° datasets, respectively, and 127 were used in all three cases. This is a subtle but important indication that crystal diffraction quality and the ability of datasets to scale together are not always correlated.

We found that there were limitations on how small an oscillation dataset could be before running into scaling problems. Key to this issue was the number of reflections in each dataset. In the case of lysozyme and CO-SWMb, which have smaller unit cells, 1° oscillation gave an insufficient number of reflections (<200) per dataset to calculate accurate scaling factors; many datasets did not have reflections in common with the others and *XSCALE* failed. Nonetheless, collecting lysozyme with 3° oscillation and CO-SWMb at 5° oscillation gave hundreds to thousands of reflections per dataset, which was sufficient to generate accurate scaling factors. We believe that this is a limitation of the processing method and we are investigating further.

Radiation damage is a major challenge in room-temperature crystallography, as the effects of damage propagate much faster at room temperature than at low temperatures. While the effects of low-dose damage can be difficult to detect in diffraction data (Owen *et al.*, 2011 ▸), it should still be possible to minimize the effects of radiation damage with fast oscillation and low angular sweeps. The dose per crystal for each chip was calculated using *RADDOSE*-3*D* (Bury *et al.*, 2018 ▸; Zeldin *et al.*, 2013 ▸). On average, 1° of exposure at 10° s^−1^ led to ∼70 kGy of dose per crystal. This is tolerable for room temperature, but longer exposures, particularly on crystals with radiation-sensitive metals like the iron-containing CO-SWMb, may lead to significant radiation damage. To probe the effects of radiation damage, we reprocessed data from the 3° FAcD dataset (Chip 2) and the 5° CO-MbCO dataset.

FAcD lacks any moieties such as disulfides that are especially prone to site-specific radiation damage so we expected that any radiation damage occurring would be stochastic in nature. We compared the first 1° and last 1° datasets from Chip 2, processed as described above. The data-processing statistics and refinement data are shown in Section S3 of the Supporting information. There is negligible difference in the processing statistics and Wilson *B* factors for the two datasets. When the two structures were overlaid and compared using *GESAMT* (Krissinel, 2012 ▸), they were found to have a high correlation and a low root-mean-square deviation (RMSD) of 0.072 Å indicating little to no geometric distortion as a result of radiation damage, in agreement with an experiment described previously (Schulz *et al.*, 2018 ▸).

**Figure 4:**
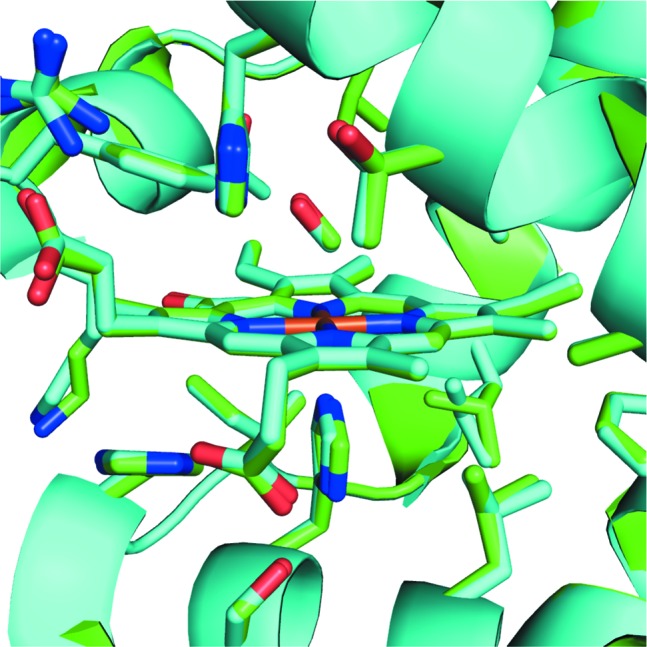
Superimposed structures of CO-SWMb data collected in the first 2° (green) and last 2° (blue), centered around the heme atom, detailing structural variations between the two models.

Crystals of CO-SWMb, on the other hand, are more prone to radiation damage because of the iron in the heme moiety, but our method did not lead to appreciable changes in structure after dose. To study this, we collected crystals of CO-SWMb at 5° oscillation – far more than needed per crystal – to effectively ‘burn’ each crystal with a dose of 197 kGy. We then compared datasets comprising the first 2° of oscillation per crystals and the last 2° of oscillation per crystal that were processed, filtered, scaled and merged separately as described in Section 2.6. The data collection and refinement statistics are shown in Section S4 of the Supporting information. The differences in data quality are, surprisingly, quite small. The second dataset has higher overall *B* factors than the first (48.6 and 40.7 Å^2^, respectively) and the cutoff, given by the CC cutoff calculated by *XSCALE*, is slightly higher (2.1 and 2.0 Å, respectively). Refinement statistics for both datasets are similar. Overlay and analysis with *GESAMT* gives an RMSD of 0.176 Å. The active heme site shows some small conformational changes in the side chains around the heme-CO moiety, as seen in Fig. 4 ▸. The N_His93_—Fe_heme_ bond is shortened upon exposure from 2.09 to 1.98 Å, and the Fe_heme_—CO bond is shortened from 2.20 to 2.14 Å. Additionally, the Fe—C—O bond angle is reduced from 136.1 to 132.5°.

It is interesting to note that this study is, effectively, time-resolved oscillation: data collection at continuous dose, followed by time-specific processing, giving an average ‘snapshot’ of the crystal state after a finite amount of time. In this way, one could envisage this to be a method for time-resolved dynamics experiments for pathways that happen on the timescale of milliseconds, complementary to the ‘hit-and-return’ time-resolved SSX system we recently described (Schulz *et al.*, 2018 ▸).

The number of diffracted X-rays scales linearly with the volume of the irradiated crystal. Thus, for a given dose near the radiation-damage limit, very small crystals yield few diffracted X-rays. The crystals used in the present study were relatively large. However, it is important to note that the use of much smaller crystals should be feasible. This would require silicon chips with smaller wells and thinner moisture-retaining windows, both of which are possible, and paying even greater attention to background reduction. Recently developed sparse data techniques (Lan *et al.*, 2018 ▸) allow the analysis of complete datasets even in cases where the number of diffracted X-rays per crystal is too few to determine the crystal orientation. Thus, the practical lower limit of crystal size using chip-based methods, such as described here, has yet to be determined.

## 4. Conclusion   

High-throughput data collection in protein crystallography, whether for serial, time-resolved or room-temperature structural studies, will become increasingly predominant as structural biology looks at more complex targets which require several complementary techniques. While electron-based methods have become the mainstay for high-profile targets in the past five years, crystallographers have a need to understand the role that crystallography will play in structural biology in the future (Grimes *et al.*, 2018 ▸). Crystallography at room temperature will become even more necessary as the limitations of low-temperature biology start to become better understood. Protein dynamics, especially, can be served well by room-temperature crystallographic methods, as the recent success of XFEL-based crystallography has made abundantly clear. But the study of protein dynamics that happen over the course of milliseconds – conformational changes, ligand binding *etc.* – can be served well by modern storage-ring sources.

The high-throughput, fixed-target serial oscillation crystallography method described here is a practical, fast and economical means to collect high-quality room-temperature diffraction data with minimized background scattering. The microchip-well technology limits the multicrystal diffraction and crystal overlap that plagues fixed-target approaches to sample delivery. Since it requires far fewer crystals to generate complete datasets than collecting still images, it is attractive in cases where one only has a small number of crystals. Future studies will be aimed at applying the technique to difficult microcrystals and time-resolved studies.

### 4.1. Raw data   

The datasets for FAcD, 1° oscillation (https://doi.org/10.5281/zenodo.2539519) and lysozyme (https://doi.org/10.5281/zenodo.2539641) are available for download from Zenodo. Because of size constraints, please contact the authors for the raw data for FAcD, 3° oscillation and for CO-SWMb.

## Supplementary Material

Supporting information- additional methods, statistics. DOI: 10.1107/S2052252519001453/mf5030sup1.pdf

Supporting video of the goniometer oscillation. DOI: 10.1107/S2052252519001453/mf5030sup2.mp4

PDB reference: 6mv0, CO-bound sperm whale myoglobin, 5° oscillation

PDB reference: 6muy, fluoro­acetate dehalogenase, 3° oscillation

PDB reference: 6muh, fluoro­acetate dehalogenase, 1° oscillation

PDB reference: 6muz, lysozyme, 3° oscillation

Dataset for FAcD, 1 degree oscillation URL: https://doi.org/10.5281/zenodo.2539519

Dataset for lysozyme URL: https://doi.org/10.5281/zenodo.2539641

## Figures and Tables

**Table 1:** Data-collection parameters and refinement statistics Statistics for the outermost resolution shell are in parentheses.

	FAcD, Chip 1	FAcD, Chip 2	Lysozyme	CO-SWMb
Oscillation angle (°)	1°	3°	3°	5°
Oscillation velocity (° s^−1^)	10	10	10	10
Wavelength (Å)	1.216	1.216	1.216	1.216
Absorbed dose (kGy)†	68.2	147.9	169.3	303.1
Dose per degree at 10° s^−1^	40.7	40.7	46.6	54.4
Wells collected	6400	13411	4040	6400
Datasets with crystals (hit %)‡	3293 (48.5%)	2626 (19.6%)	1003 (24.8%)	2710 (42.3%)
Indexable datasets	1554	2269	510	830
Number of datasets used in refinement	494	249	95	138
Space group	*P*2_1_	*P*2_1_	*P*4_3_2_1_2	*P*2_1_2_1_2_1_
Unit cell	41.6, 79.1, 83.8, 90, 103, 90	41.6, 79.1, 83.8, 90, 103, 90	79.1, 79.1, 38.02, 90, 90, 90	37.03, 45.9, 82.91, 90, 90, 90
Resolution range (Å)	33.43–1.8 (1.864–1.8)	39.55–1.8 (1.864–1.8)	39.55–1.839 (1.905–1.839)	40.16–1.97 (2.04–1.97)
Total reflections	306210 (23803)	613807 (45550)	182350 (12878)	236292 (21259)
Unique reflections	48288 (4597)	48608 (4680)	10841 (981)	10477 (1006)
Multiplicity	6.3 (5.2)	12.6 (9.7)	16.8 (13.1)	22.6 (21.1)
Completeness (%)	98.42 (94.72)	99.02 (96.37)	98.87 (90.17)	99.83 (99.41)
Mean *I*/σ(*I*)	9.33 (1.60)	6.65 (0.53)	10.01 (2.68)	10.93 (0.73)
Wilson *B* factor (Å)^2^	27.55	34.40	23.13	42.37
*R*_meas_	0.188 (3.651)	0.2694 (8.233)	0.3388 (7.666)	0.2541 (16.48)
*R*_p.i.m_	0.07252 (1.554)	0.07347 (2.599)	0.08115 (2.037)	0.05271 (3.53)
CC_1/2_	0.993 (0.176)	0.992 (0.106)	0.991 (0.352)	0.994 (0.181)
CC*	0.998 (0.548)	0.998 (0.437)	0.998 (0.722)	0.999 (0.554)
Reflections used in refinement	48270 (4592)	48559 (4676)	10840 (981)	10472 (1006)
Reflections used for *R*_free_	2413 (230)	2422 (236)	541 (49)	524 (50)
*R*_work_/*R*_free_	0.1518/0.1812 (0.3591/0.3796)	0.1631/0.1984 (0.4598/0.5082)	0.1703/0.2043 (0.2961/0.3054)	0.1976/0.2731 (0.3943/0.3851)
Number of non-H atoms	4976	4972	1058	1299
Macromolecules	4765	4754	1016	1225
Ligands	0	0	3	50
Solvent	211	217	39	24
Protein residues	596	595	129	155
RMS (bonds) (Å)	0.008	0.008	0.007	0.009
RMS (angles) (°)	1.30	1.29	1.15	1.24
Ramachandran plot				
Favored (%)	97.47	97.29	98.43	95.39
Allowed (%)	2.53	2.54	1.57	4.61
Outliers (%)	0.00	0.17	0	0.00
Rotamer outliers (%)	0.42	0.63	0	0.00
Clashscore	4.90	7.16	2.0	7.43
Average *B* factor (Å^2^)	34.42	40.26	26.16	50.67
Macromolecules	34.14	40.00	25.97	50.66
Ligands	—	—	29.87	48.61
Solvent	40.63	45.82	31.01	55.90
Number of TLS groups	15	17	9	4
PDB codes	6muh	6muy	6muz	6mvo

† This value includes the initial 0.5° oscillation that precedes data collection.

‡ ‘Hit’ is defined as a dataset that had sufficient spots for *XDS* to attempt indexing.
