# Supplementary material for: Fixed-target serial oscillation crystallography at room temperature
Source: IUCrJ. 2019 Feb 23;6(Pt 2):305–16. doi: 10.1107/S2052252519001453 (PMC6400179; doi:10.1107/S2052252519001453)
Supplement: Supplementary file 1 [file m-06-00305-sup1.pdf]

IUCrJ

Volume 6 (2019)

Supporting information for article:

Fixed-target serial oscillation crystallography at room temperature

Jennifer L. Wierman, Olivier Paré-Labrosse, Antoine Sarracini, Jessica Besaw, Michael J. Cook, Saeed Oghbaey, Hazem Daoud, Pedram Mehrabi, Irina Kriksunov, Anling Kuo, David J. Schuller, Scott Smith, Oliver P. Ernst, Doletha M. E. Szebenyi, Sol M. Gruner, R. J. Dwayne Miller and Aaron D. Finke

Supporting Information

S1. Sperm Whale Myoglobin Expression and Purification

SW myoglobin(Mb) plasmid DNA (pMb412a- D122N) is a gift from Dr. John S. Olson's lab (Rice University, USA), (Springer, B. A.; Sligar, 1987). The plasmid was freshly transformed into BL21(DE)3 cells. The protein was expressed constitutively in the *E. coli* strain BL21(DE3) at 37°C for three days. The cells were harvested and the pellets were washed once in 1x Tris Buffered Saline (TBS, Fisher BioReagents), and were stored at -80°C until further use.

SWMb was purified as described (Mueller, C. et al. and Springer and Sligar) with some modifications. All purification buffers were saturated with 1 atm of CO gas before use. The initial ammonium sulfate percentage saturation was ~60% at 0°C. Subsequently, the supernatant was saturated with ammonium sulfate at 0°C. After precipitation, the pellet was dissolved in 10-15 ml of 20 mM sodium-phosphate pH 6.0 and dialyzed overnight against the same buffer at 4°C (~1 to 2 L of CO saturated buffer. The dialyzed protein was centrifuged at 80,000 *g* for 30 min. and the supernatant was applied to a CM Sepharose (GE Healthcare Life Science) fast flow column which was pre-equilibrated with the same buffer and a linear pH gradient (20 mM sodium-phosphate pH 6.0 to 50 mM sodium-phosphate pH 9.3) was used for elution under 1 atm of CO. The eluted protein fractions were concentrated and applied to a Superdex 75 gel filtration column (GE Healthcare Life Science) in 20 mM Tris-HCl and 150 mM NaCl buffer pH 8.0 (CO saturated). The peak fractions were combined and concentrated to 15-20 mg/ml. Solid sodium hydrosulfite was added at a final concentration of 20 mM. After 10-15 min. incubation, the protein was rapidly applied to a Sephadex G-25 column equilibrated with 10 mM Tris-HCl buffer (pH 9.0) under 1 atm of CO. The eluate containing SWMb-CO was concentrated to ~60 mg/ml for crystallization.

S2. XSCALE Processing results for all Datasets

Defluorinase Chip 1, 1° oscillation

data from 2269 crystals collected

data from 494 crystals merged and used in refinement

SUBSET OF INTENSITY DATA WITH SIGNAL/NOISE >= -3.0 AS FUNCTION OF RESOLUTION

RESOLUTION LIMIT	NUMBER OF REFLECTIONS OBSERVED	UNIQUE	POSSIBLE	COMPLETENESS OF DATA	R-FACTOR observed	R-FACTOR expected	COMPARED	I/SIGMA	R-meas	CC(1/2)	Anomal Corr	SigAno	Nano
8.01	3806	577	592	97.5%	9.5%	8.2%	3795	24.70	10.4%	99.0*	0	0.935	351
5.67	6260	1029	1041	98.8%	8.4%	8.3%	6227	22.20	9.2%	98.6*	-2	0.893	624
4.63	8098	1313	1317	99.7%	8.3%	8.3%	8070	22.27	9.0%	99.0*	0	0.860	800
4.01	9823	1587	1601	99.1%	7.8%	8.3%	9777	22.48	8.5%	99.3*	0	0.817	977
3.58	11408	1756	1768	99.3%	8.2%	8.8%	11382	21.27	8.9%	99.4*	-1	0.816	1135
3.27	12844	1964	1977	99.3%	9.6%	9.7%	12808	19.22	10.5%	99.0*	0	0.856	1298
3.03	13880	2111	2118	99.7%	11.7%	11.3%	13846	16.62	12.8%	99.0*	0	0.876	1402
2.83	15077	2282	2299	99.3%	14.4%	13.8%	15031	14.26	15.6%	98.9*	-3	0.839	1561
2.67	16063	2426	2436	99.6%	18.2%	17.3%	16031	12.27	19.8%	98.0*	2	0.877	1662
2.53	16853	2564	2592	98.9%	22.8%	21.2%	16830	10.84	24.8%	97.4*	0	0.877	1774
2.42	17501	2660	2677	99.4%	27.4%	26.1%	17454	9.50	29.7%	96.8*	-2	0.852	1799
2.31	18185	2787	2803	99.4%	34.0%	32.6%	18150	8.43	37.0%	95.3*	3	0.880	1875
2.22	19136	2938	2975	98.8%	45.9%	44.2%	19097	7.17	49.9%	90.7*	-1	0.847	1966
2.14	19939	3023	3061	98.8%	58.8%	56.7%	19897	6.07	63.9%	88.9*	0	0.834	2053
2.07	20253	3116	3154	98.8%	75.1%	75.6%	20211	5.12	81.8%	81.7*	1	0.806	2130
2.00	20889	3248	3299	98.5%	100.7%	101.9%	20845	4.17	109.6%	76.1*	0	0.788	2142
1.94	20822	3289	3352	98.1%	142.0%	145.7%	20781	3.34	154.8%	60.6*	0	0.758	2164
1.89	21380	3357	3450	97.3%	192.7%	203.6%	21332	2.67	210.0%	51.4*	3	0.733	2222
1.84	20485	3508	3600	97.4%	279.9%	298.1%	20431	1.95	308.0%	29.4*	-3	0.689	2103
1.79	13768	2883	3646	79.1%	385.0%	424.6%	13501	1.41	430.8%	18.5*	3	0.659	1282
total	306470	48418	49758	97.3%	17.3%	17.2%	305496	9.31	18.8%	99.3*	0	0.814	31320

Defluorinase Chip 2, 3° oscillation

Data from 1526 crystals collected

Data from 249 crystals merged and used in refinement

SUBSET OF INTENSITY DATA WITH SIGNAL/NOISE >= -3.0 AS FUNCTION OF RESOLUTION

RESOLUTION LIMIT	NUMBER OF REFLECTIONS OBSERVED	UNIQUE	POSSIBLE	COMPLETENESS OF DATA	R-FACTOR observed	R-FACTOR expected	COMPARED	I/SIGMA	R-meas	CC(1/2)	Anomal Corr	SigAno	Nano
7.97	7568	601	604	99.5%	13.0%	12.7%	7565	22.52	13.5%	99.1*	9	1.006	430
5.64	12372	1048	1055	99.3%	14.3%	13.9%	12355	19.43	14.9%	98.9*	1	0.903	744
4.60	16427	1336	1345	99.3%	14.7%	13.9%	16418	19.58	15.4%	98.9*	-1	0.941	1039
3.99	19675	1604	1611	99.6%	14.9%	14.0%	19670	19.70	15.6%	98.6*	2	0.981	1270
3.56	23021	1818	1823	99.7%	16.1%	15.0%	23009	17.89	16.8%	99.1*	-2	0.963	1499
3.25	25198	1986	1989	99.8%	19.0%	17.5%	25191	15.32	19.8%	98.8*	1	0.955	1652
3.01	28280	2159	2160	100.0%	23.2%	21.7%	28276	12.64	24.0%	98.2*	3	0.947	1827
2.82	30278	2320	2326	99.7%	28.0%	27.3%	30274	10.23	29.1%	98.0*	1	0.898	1940
2.66	32659	2485	2497	99.5%	36.1%	36.1%	32650	8.34	37.5%	96.7*	2	0.863	2143
2.52	34481	2601	2612	99.6%	44.1%	44.9%	34477	7.09	45.8%	95.4*	2	0.838	2253
2.40	36333	2725	2747	99.2%	53.9%	56.1%	36323	5.92	55.9%	93.5*	3	0.826	2376
2.30	37521	2817	2831	99.5%	65.8%	69.6%	37514	5.01	68.2%	91.3*	3	0.802	2432
2.21	39464	2976	2990	99.5%	86.8%	94.4%	39460	4.06	90.1%	79.3*	3	0.814	2584
2.13	41242	3099	3122	99.3%	108.1%	119.9%	41236	3.33	112.2%	80.9*	3	0.773	2733
2.06	42516	3209	3238	99.1%	143.4%	163.7%	42506	2.51	148.9%	74.2*	-1	0.726	2816
1.99	42735	3238	3272	99.0%	191.8%	223.6%	42729	1.98	199.1%	63.4*	0	0.683	2858
1.93	43766	3435	3475	98.8%	284.6%	342.0%	43750	1.41	295.8%	44.9*	0	0.642	2971
1.88	43225	3465	3512	98.7%	477.1%	599.3%	43214	0.92	496.6%	26.0*	2	0.587	2975
1.83	38837	3577	3622	98.8%	662.0%	850.9%	38813	0.62	693.5%	16.5*	0	0.552	2932
1.78	19048	2427	3751	64.7%	1100.8%	1461.0%	18795	0.43	1167.8%	9.4	1	0.511	1499
total	614646	48926	50582	96.7%	27.0%	27.7%	614225	6.61	28.0%	99.2*	2	0.774	40973

Defluorinase Chip 2, first 1° oscillation

Data from 1082 crystals collected

Data from 394 crystals merged and used in refinement

SUBSET OF INTENSITY DATA WITH SIGNAL/NOISE >= -3.0 AS FUNCTION OF RESOLUTION

RESOLUTION LIMIT	NUMBER OF REFLECTIONS OBSERVED	UNIQUE	POSSIBLE	COMPLETENESS OF DATA	R-FACTOR observed	R-FACTOR expected	COMPARED	I/SIGMA	R-meas	CC(1/2)	Anomal Corr	SigAno	Nano
7.81	3615	622	635	98.0%	13.8%	13.1%	3599	18.10	15.1%	98.8*	1	0.905	335
5.52	5986	1119	1131	98.9%	15.6%	14.2%	5940	14.69	17.2%	97.1*	-6	0.920	566
4.51	7843	1419	1445	98.2%	15.8%	13.9%	7794	14.96	17.5%	85.6*	0	0.922	719
3.90	9370	1685	1699	99.2%	16.7%	14.8%	9308	14.54	18.3%	97.4*	1	1.007	895
3.49	10754	1882	1904	98.8%	18.0%	15.9%	10695	12.92	19.7%	97.1*	3	0.972	1048
3.19	12420	2101	2127	98.8%	21.8%	19.4%	12347	10.69	23.9%	96.2*	5	0.969	1217
2.95	13800	2298	2319	99.1%	27.5%	25.4%	13750	8.51	30.1%	95.6*	0	0.890	1380
2.76	14713	2443	2458	99.4%	35.0%	34.4%	14641	6.59	38.2%	92.9*	3	0.855	1450
2.60	15892	2629	2638	99.7%	45.2%	46.9%	15827	5.30	49.3%	90.7*	2	0.787	1573
2.47	16611	2741	2768	99.0%	56.8%	60.8%	16551	4.43	62.1%	86.6*	3	0.767	1671
2.35	17402	2907	2926	99.4%	72.6%	80.0%	17316	3.58	79.3%	82.1*	-1	0.729	1719
2.25	18347	3035	3056	99.3%	95.2%	108.9%	18274	2.95	104.0%	65.0*	3	0.726	1824
2.17	19176	3153	3180	99.2%	126.5%	147.7%	19098	2.37	138.0%	63.2*	-2	0.690	1906
2.09	19488	3263	3279	99.5%	159.9%	198.2%	19400	1.81	174.8%	49.1*	0	0.662	1951
2.02	20255	3413	3448	99.0%	243.3%	297.8%	20173	1.34	266.3%	33.4*	2	0.634	2042

1.95	20170	3480	3513	99.1%	405.3%	518.1%	20088	1.00	445.9%	5.0	0	0.592	2029
1.89	20566	3595	3633	99.0%	805.9%	1079.1%	20460	0.68	884.7%	7.5	1	0.557	2054
1.84	19467	3719	3776	98.5%	456.4%	590.5%	19314	0.42	504.4%	4.3	1	0.534	1923
1.79	12088	2988	3850	77.6%	756.4%	1018.6%	11652	0.27	852.6%	0.2	-1	0.503	1086
1.75	71	70	3948	1.8%	-99.9%	-99.9%	2	0.00	-99.9%	0.0	0	0.000	0
total	278034	48562	53733	90.4%	37.7%	40.8%	276229	4.69	41.3%	95.2*	1	0.729	27388

Defluorinase Chip 2, last 1° oscillation

Data from 1082 crystals collected

Data from 428 crystals merged and used in refinement

SUBSET OF INTENSITY DATA WITH SIGNAL/NOISE >= -3.0 AS FUNCTION OF RESOLUTION

RESOLUTION LIMIT	NUMBER OF REFLECTIONS OBSERVED	UNIQUE	POSSIBLE	COMPLETENESS OF DATA	R-FACTOR observed	R-FACTOR expected	COMPARED	I/SIGMA	R-meas	CC(1/2)	Anomal Corr	SigAno	Nano
7.51	4280	703	717	98.0%	15.0%	14.1%	4259	17.74	16.4%	96.3*	10	0.968	358
5.31	7139	1238	1253	98.8%	17.6%	16.0%	7096	13.97	19.4%	93.6*	-2	0.964	640
4.34	9595	1621	1643	98.7%	16.5%	15.2%	9539	14.89	18.1%	93.7*	3	0.964	916
3.75	11328	1858	1891	98.3%	18.2%	16.3%	11291	13.59	19.9%	97.8*	-2	0.919	1122
3.36	13243	2148	2167	99.1%	22.3%	19.5%	13203	11.43	24.5%	74.4*	3	0.988	1297
3.07	14868	2374	2395	99.1%	27.9%	25.4%	14803	8.68	30.4%	95.0*	-2	0.878	1433
2.84	16290	2581	2585	99.8%	37.4%	35.2%	16237	6.52	40.7%	91.9*	0	0.873	1584
2.65	17769	2776	2792	99.4%	50.6%	50.7%	17720	5.04	55.0%	89.7*	2	0.822	1800
2.50	19009	2926	2945	99.4%	67.7%	69.4%	18940	4.13	73.5%	82.4*	1	0.807	1924
2.37	20191	3105	3128	99.3%	88.9%	95.2%	20119	3.20	96.4%	73.4*	-2	0.749	1995
2.26	21082	3237	3262	99.2%	120.0%	132.9%	21031	2.59	130.3%	54.8*	5	0.732	2100
2.17	21909	3407	3428	99.4%	167.5%	186.5%	21837	2.01	182.1%	45.2*	1	0.688	2209
2.08	22500	3523	3535	99.7%	250.4%	286.2%	22435	1.50	272.5%	14.4*	1	0.661	2273
2.01	23099	3724	3755	99.2%	300.8%	359.8%	23024	1.13	328.0%	16.8*	1	0.631	2352
1.94	23276	3835	3864	99.2%	636.9%	761.4%	23180	0.76	695.7%	10.6*	0	0.599	2365
1.88	23205	3917	3954	99.1%	1093.2%	1285.7%	23101	0.47	1194.8%	8.4	3	0.543	2297
1.82	21403	4028	4071	98.9%	2230.1%	2638.6%	21214	0.28	2464.7%	-1.2	-1	0.527	2094
1.77	5868	1646	4257	38.7%	2065.0%	2407.7%	5466	0.15	2343.5%	-3.3	8	0.516	454
1.72	30	30	4275	0.7%	-99.9%	-99.9%	0	0.00	-99.9%	0.0	0	0.000	0
1.68	8	8	4467	0.2%	-99.9%	-99.9%	0	0.00	-99.9%	0.0	0	0.000	0
total	296092	48685	60384	80.6%	41.1%	42.6%	294495	4.27	44.9%	94.1*	1	0.732	29213

CO-SWMb, 5° oscillation

Data from 834 crystals collected

Data from 138 Crystals merged and used in refinement

SUBSET OF INTENSITY DATA WITH SIGNAL/NOISE >= -3.0 AS FUNCTION OF RESOLUTION

RESOLUTION LIMIT	NUMBER OF REFLECTIONS OBSERVED	UNIQUE	POSSIBLE	COMPLETENESS OF DATA	R-FACTOR observed	R-FACTOR expected	COMPARED	I/SIGMA	R-meas	CC(1/2)	Anomal Corr	SigAno	Nano
8.14	3497	275	276	99.6%	10.8%	10.3%	3496	29.00	11.3%	99.5*	57*	1.467	91
5.76	5834	494	495	99.8%	10.2%	10.9%	5833	23.75	10.7%	99.5*	33*	1.193	198
4.70	8021	657	657	100.0%	10.8%	11.2%	8021	24.04	11.2%	99.5*	29*	1.174	280
4.07	9311	765	765	100.0%	10.3%	11.1%	9311	23.52	10.7%	99.4*	19	1.069	330
3.64	10618	860	860	100.0%	11.5%	11.9%	10618	21.40	12.0%	99.5*	16	1.012	383
3.32	12219	970	971	99.9%	13.9%	13.8%	12219	17.87	14.4%	99.5*	20*	1.122	435
3.08	12859	1028	1028	100.0%	20.1%	19.0%	12859	13.63	21.0%	98.9*	14	1.071	466
2.88	14094	1121	1122	99.9%	31.0%	28.9%	14094	9.81	32.3%	97.7*	10	1.075	512
2.71	15171	1205	1207	99.8%	44.8%	41.4%	15170	7.56	46.7%	95.5*	15	1.079	549
2.57	15939	1261	1268	99.4%	57.4%	53.5%	15939	6.15	59.8%	93.8*	11	1.074	577
2.45	16605	1320	1329	99.3%	86.1%	80.1%	16605	4.58	89.8%	85.2*	10	1.043	606
2.35	17436	1390	1393	99.8%	104.3%	97.3%	17436	3.82	108.7%	81.5*	12	1.066	644
2.26	18112	1445	1450	99.7%	148.0%	139.2%	18112	2.80	154.2%	69.5*	8	0.974	668
2.18	18487	1469	1482	99.1%	215.9%	205.8%	18486	2.07	224.9%	54.2*	10	0.973	679
2.10	19082	1558	1581	98.5%	321.1%	313.2%	19082	1.42	335.1%	38.2*	8	0.860	723
2.03	19178	1593	1615	98.6%	498.8%	487.4%	19175	0.89	520.9%	23.5*	5	0.760	731
1.97	18350	1613	1657	97.3%	1583.0%	1597.5%	18348	0.51	1657.6%	14.6*	2	0.667	739
1.92	19094	1725	1746	98.8%	10684.9%	11089.1%	19092	0.26	11203.5%	8.5	3	0.595	799
1.87	17876	1733	1770	97.9%	15682.9%	16753.7%	17868	0.11	16504.6%	-0.0	-4	0.526	790
1.82	10789	1398	1794	77.9%	3282.9%	3426.9%	10642	0.04	3492.5%	5.0	-2	0.546	554
total	282572	23880	24466	97.6%	28.4%	28.0%	282406	6.48	29.6%	99.6*	10	0.900	10754

CO-SWMb, first 2° oscillation

Data from 723 crystals collected

Data from 309 crystals merged and used in refinement

SUBSET OF INTENSITY DATA WITH SIGNAL/NOISE >= -3.0 AS FUNCTION OF RESOLUTION

RESOLUTION LIMIT	NUMBER OF REFLECTIONS OBSERVED	UNIQUE	POSSIBLE	COMPLETENESS OF DATA	R-FACTOR observed	R-FACTOR expected	COMPARED	I/SIGMA	R-meas	CC(1/2)	Anomal Corr	SigAno	Nano
8.14	2726	275	276	99.6%	12.1%	11.3%	2725	25.13	12.7%	99.3*	84*	1.392	91
5.76	4387	495	495	100.0%	13.8%	13.2%	4384	18.84	14.7%	98.4*	22	1.103	198
4.70	6063	657	657	100.0%	14.3%	13.3%	6063	19.14	15.1%	99.3*	22*	1.076	280
4.07	7170	765	765	100.0%	14.0%	13.6%	7170	18.96	14.8%	99.2*	18	1.085	330
3.64	8136	860	860	100.0%	15.1%	14.4%	8134	16.65	16.0%	99.1*	16	1.013	382
3.32	9531	970	971	99.9%	19.2%	18.0%	9531	13.49	20.3%	99.0*	8	0.989	434
3.08	9991	1026	1030	99.6%	27.4%	27.0%	9989	9.81	28.9%	97.8*	10	0.905	462
2.88	10932	1118	1122	99.6%	44.3%	43.8%	10929	6.85	46.8%	95.0*	8	0.933	507
2.71	11746	1201	1205	99.7%	62.9%	64.7%	11745	5.13	66.4%	90.2*	9	0.911	546

2.57	12356	1262	1268	99.5%	80.5%	84.4%	12354	4.17	84.9%	85.9*	8	0.873	577
2.45	12781	1318	1333	98.9%	121.3%	131.5%	12779	3.02	128.1%	69.5*	5	0.804	603
2.35	13230	1377	1389	99.1%	144.2%	157.9%	13229	2.53	152.3%	66.4*	9	0.829	635
2.26	13610	1429	1450	98.6%	218.9%	245.8%	13605	1.91	231.4%	50.5*	7	0.768	650
2.18	13733	1458	1484	98.2%	308.3%	352.7%	13732	1.58	326.1%	33.3*	0	0.725	669
2.10	13911	1554	1581	98.3%	584.0%	674.0%	13909	0.99	620.0%	11.2*	8	0.705	717
2.03	14346	1586	1617	98.1%	778.8%	930.3%	14339	0.77	826.1%	12.6*	3	0.638	722
1.97	14205	1605	1653	97.1%	913.9%	1096.5%	14199	0.49	970.6%	3.5	-2	0.587	728
1.92	14767	1693	1750	96.7%	2693.2%	3268.5%	14766	0.42	2863.5%	0.6	2	0.560	769
1.87	14482	1712	1766	96.9%	8147.1%	9874.3%	14478	0.25	8675.7%	2.8	3	0.500	778
1.82	8204	1310	1796	72.9%	3635.6%	4439.9%	8069	0.14	3936.5%	-0.0	7	0.535	511
total	216307	23671	24468	96.7%	40.8%	44.1%	216129	4.95	43.3%	99.1*	10	0.774	10589

CO-SWMb, last 2° oscillation

Data from 909 datasets collected

Data from 299 crystals merged and used in refinement

SUBSET OF INTENSITY DATA WITH SIGNAL/NOISE >= -3.0 AS FUNCTION OF RESOLUTION

RESOLUTION LIMIT	NUMBER OF REFLECTIONS OBSERVED	UNIQUE	POSSIBLE	COMPLETENESS OF DATA	R-FACTOR observed	R-FACTOR expected	COMPARED	I/SIGMA	R-meas	CC(1/2)	Anomal Corr	SigAno	Nano
8.94	1977	206	207	99.5%	13.5%	11.5%	1975	26.94	14.3%	99.4*	82*	1.300	66
6.32	3537	372	373	99.7%	11.3%	11.0%	3536	22.39	12.0%	99.6*	25	1.125	147
5.16	4321	483	484	99.8%	14.0%	13.2%	4320	18.45	14.9%	99.2*	25	1.152	201
4.47	5396	584	586	99.7%	13.1%	12.4%	5393	19.54	13.9%	99.4*	5	1.000	245
4.00	6145	655	657	99.7%	13.9%	12.9%	6145	19.21	14.7%	99.2*	21*	1.087	288
3.65	6789	712	714	99.7%	15.2%	14.3%	6787	16.85	16.0%	99.2*	19	1.010	313
3.38	7560	793	795	99.7%	17.3%	16.4%	7558	14.42	18.3%	98.7*	18	0.962	353
3.16	8200	854	856	99.8%	27.6%	26.4%	8199	9.76	29.2%	97.6*	10	0.978	385
2.98	8664	897	900	99.7%	38.0%	37.0%	8663	7.54	40.2%	95.6*	5	1.009	406
2.83	9121	932	937	99.5%	48.1%	49.7%	9119	5.76	50.8%	95.4*	6	0.910	422
2.70	9633	1005	1011	99.4%	68.4%	71.9%	9632	4.50	72.3%	88.8*	-3	0.848	461
2.58	10071	1048	1054	99.4%	83.9%	88.0%	10070	3.70	88.6%	83.7*	14	0.953	477
2.48	10278	1079	1090	99.0%	128.7%	138.0%	10276	2.54	136.0%	66.8*	10	0.879	493
2.39	10678	1129	1142	98.9%	132.4%	144.9%	10674	2.39	140.0%	62.5*	6	0.868	514
2.31	10859	1147	1163	98.6%	187.5%	206.9%	10857	1.79	198.2%	53.2*	2	0.826	524
2.24	11220	1230	1250	98.4%	259.4%	290.4%	11220	1.38	275.0%	38.6*	-7	0.730	562
2.17	11065	1201	1223	98.2%	349.8%	404.1%	11064	1.06	370.4%	24.3*	-2	0.732	549
2.11	11474	1278	1304	98.0%	491.4%	577.0%	11470	0.74	521.4%	15.4*	2	0.691	585
2.05	11365	1317	1343	98.1%	677.0%	818.6%	11362	0.55	720.0%	7.1	0	0.614	601
2.00	11573	1345	1372	98.0%	1293.7%	1593.7%	11570	0.34	1376.8%	-0.8	-2	0.607	616
total	169926	18267	18461	98.9%	26.2%	26.7%	169890	6.11	27.8%	99.5*	8	0.851	8208

Lysozyme, 3° oscillation

Data from 385 crystals collected

Data from 95 crystals merged and used in refinement

SUBSET OF INTENSITY DATA WITH SIGNAL/NOISE >= -3.0 AS FUNCTION OF RESOLUTION

RESOLUTION LIMIT	NUMBER OF REFLECTIONS OBSERVED	UNIQUE	POSSIBLE	COMPLETENESS OF DATA	R-FACTOR observed	R-FACTOR expected	COMPARED	I/SIGMA	R-meas	CC(1/2)	Anomal Corr	SigAno	Nano
8.23	2397	160	162	98.8%	14.8%	13.9%	2396	20.57	15.3%	98.5*	13	0.987	71
5.82	3819	249	250	99.6%	14.1%	14.4%	3817	19.78	14.6%	98.7*	9	0.909	156
4.75	4997	315	315	100.0%	14.5%	14.8%	4995	19.29	15.0%	98.9*	11	0.939	219
4.11	5899	364	364	100.0%	14.1%	14.5%	5899	20.25	14.5%	99.2*	5	0.922	267
3.68	6861	407	407	100.0%	14.5%	14.9%	6861	19.89	15.0%	99.1*	5	0.907	310
3.36	7495	441	441	100.0%	16.4%	16.3%	7495	18.03	16.9%	99.2*	-1	0.902	343
3.11	8162	473	473	100.0%	19.1%	18.6%	8162	16.52	19.7%	99.0*	5	0.945	379
2.91	8729	501	501	100.0%	24.1%	23.2%	8729	14.11	24.8%	98.5*	-1	0.924	405
2.74	9265	534	534	100.0%	29.1%	28.4%	9265	12.38	30.0%	98.1*	-2	0.867	438
2.60	10195	572	572	100.0%	34.7%	33.0%	10194	11.58	35.7%	98.0*	0	0.899	476
2.48	10261	592	593	99.8%	43.9%	41.5%	10261	10.23	45.3%	97.3*	-3	0.933	495
2.37	10918	615	618	99.5%	52.6%	49.7%	10918	9.59	54.2%	95.1*	1	0.928	520
2.28	11393	650	650	100.0%	63.1%	59.3%	11393	8.63	64.9%	95.4*	-3	0.896	554
2.20	11523	648	650	99.7%	81.4%	78.0%	11522	7.70	83.8%	93.1*	0	0.945	560
2.12	12174	694	694	100.0%	100.1%	96.8%	12174	6.93	103.1%	91.2*	0	0.904	595
2.06	12608	716	717	99.9%	151.2%	147.7%	12607	5.93	155.7%	83.6*	2	0.915	619
2.00	12207	699	703	99.4%	220.1%	224.7%	12207	4.95	226.8%	79.0*	5	0.877	608
1.94	13282	776	778	99.7%	322.5%	333.9%	13282	4.23	332.4%	70.7*	0	0.843	674
1.89	11568	753	759	99.2%	512.1%	546.6%	11567	3.38	529.8%	55.5*	-1	0.742	653
1.84	8634	695	798	87.1%	752.0%	800.4%	8601	2.62	782.8%	27.0*	-3	0.733	539
total	182387	10854	10979	98.9%	32.9%	32.6%	182345	10.00	33.9%	99.1*	1	0.884	8881

S3. Refinement Statistics for Defluorinase Chip 2, First and Last 1° Oscillation of 3° Total Oscillation

	First 1° Oscillation	Last 1° Oscillation
Wells Collected	6400	6400
Indexable Datasets	1082	1082
No. Datasets Used in Refinement	394	428
Wavelength	1.216	1.216
Resolution range	39.55 - 1.8 (1.864 - 1.8)	33.43 - 1.9 (1.968 - 1.9)
Space group	P 1 21 1	P 1 21 1
Unit cell	41.6 79.1 83.8 90 103 90	41.6 79.1 83.8 90 103 90
Total reflections	277738 (20316)	259881 (24650)
Unique reflections	48343 (4680)	41428 (3991)
Multiplicity	5.7 (4.5)	6.3 (6.0)
Completeness (%)	99.02 (96.35)	98.98 (96.70)
Mean I/sigma(I)	4.71 (0.32)	4.97 (0.60)
Wilson B-factor	34.41	33.40
R-merge	0.3764 (7.471)	0.3694 (7.32)
R-meas	0.4132 (8.37)	0.4028 (7.99)
R-pim	0.1648 (3.646)	0.1554 (3.103)
CC1/2	0.954 (0.00274)	0.944 (0.0265)
CC*	0.988 (0.0739)	0.985 (0.227)
Reflections used in refinement	48557 (4675)	41312 (3990)
Reflections used for R-free	2442 (236)	2066 (200)
R-work	0.1746 (0.4611)	0.1778 (0.3818)
R-free	0.2105 (0.4816)	0.2239 (0.4197)
CC(work)	0.962 (0.134)	0.971 (0.443)
CC(free)	0.940 (0.331)	0.957 (0.425)
Number of non-hydrogen atoms	4972	4972
macromolecules	4754	4754
ligands	1	1
solvent	217	217
Protein residues	595	595
RMS(bonds)	0.008	0.007
RMS(angles)	1.29	0.93
Ramachandran favored (%)	97.29	96.62
Ramachandran allowed (%)	2.54	3.21
Ramachandran outliers (%)	0.17	0.17
Rotamer outliers (%)	0.63	0.63
Clashscore	8.34	8.77
Average B-factor	37.97	36.81
macromolecules	37.73	36.59
ligands	57.12	55.59
solvent	43.12	41.55
PDB Code	6MZZ	6N00

S4. Refinement Statistics for CO-SWMb, First and Last 2° Oscillation of 5° Total Oscillation

	First 2°	Last 2°
Wells Collected	6400	6400
Indexable Datasets	834	834
No. Datasets Used in Refinement	394	428
Wavelength (Å)	1.216	1.216
Resolution range	27.22 - 2.0 (2.071 - 2.0)	40.16 - 2.1 (2.175 - 2.1)
Space group	P 21 21 21	P 21 21 21
Unit cell	37.03 45.9 82.91 90 90 90	37.03 45.9 82.91 90 90 90
Total reflections	172975 (16346)	148607 (14193)
Unique reflections	10029 (989)	8687 (853)
Multiplicity	17.2 (16.5)	17.1 (16.6)
Completeness (%)	99.77 (99.70)	99.76 (99.65)
Mean I/sigma(I)	8.66 (0.90)	9.50 (1.04)
Wilson B-factor	40.70	48.59
R-merge	0.3369 (8.751)	0.2497 (4.961)
R-meas	0.3473 (9.03)	0.2576 (5.117)
R-pim	0.08235 (2.195)	0.06171 (1.234)
CC1/2	0.992 (0.153)	0.997 (0.253)
CC*	0.998 (0.515)	0.999 (0.635)
Reflections used in refinement	10029 (989)	8687 (853)
Reflections used for R-free	502 (50)	435 (42)
R-work	0.1959 (0.3855)	0.1987 (0.3781)
R-free	0.2815 (0.4147)	0.2847 (0.4049)
CC(work)	0.969 (0.402)	0.965 (0.546)
CC(free)	0.953 (0.205)	0.961 (0.574)
Number of non-hydrogen atoms	1316	1302
macromolecules	1236	1236
ligands	50	50
solvent	30	16
Protein residues	155	154
RMS(bonds)	0.009	0.008
RMS(angles)	1.23	1.02
Ramachandran favored (%)	96.71	97.37
Ramachandran allowed (%)	3.29	2.63
Ramachandran outliers (%)	0.00	0.00
Rotamer outliers (%)	0.79	0.00
Clashscore	10.85	11.62
Average B-factor	51.00	52.03
macromolecules	50.97	52.03
ligands	51.14	50.80
solvent	51.81	55.53
Number of TLS Groups	4	5
PDB Code	6N02	6N03

Sample BASH script for batch processing

```
#!/bin/bash
# does spot collection for a given directory, then processes the data
# directories that do not contain a list of HKL/intensities/sigmas will be
# deleted
# A. Finke rev 11/9/2017

for masterfile in `ls path/to/images/*master.h5`
do
    masterfilename=`echo $masterfile | awk -F "/" '{print $NF}'`
    filetemplate="${masterfilename%_master.h5}"
    datatemplate="${masterfile%_master.h5}"

    rm -rf $filetemplate
    mkdir $filetemplate
    cd $filetemplate
    cat > XDS.INP <<eof
!====================== DETECTOR PARAMETERS ==================================
 DETECTOR=EIGER
 MINIMUM_VALID_PIXEL_VALUE=0
 OVERLOAD= 1048500   ! presumably much higher 2^31
 SENSOR_THICKNESS=0.32 ! [mm]
!SILICON=-1.0
!GAIN=1.0  ! pixel contents is interpreted as X-ray counts
 QX=0.075  QY=0.075  ! [mm]
 NX= 1030  NY= 1065  !  1M
!NX= 2070  NY= 2167  !  4M
!NX= 3110  NY= 3269  !  9M
!NX= 4150  NY= 4371  ! 16M

!EXCLUSION OF VERTICAL DEAD AREAS OF THE EIGER 1M DETECTOR

!EXCLUSION OF HORIZONTAL DEAD AREAS OF THE EIGER 1M DETECTOR
 UNTRUSTED_RECTANGLE=    0 1031    514  552
!SILICON DIFFRACTION MASK
 !UNTRUSTED_RECTANGLE=    695 718 230 250

 TRUSTED_REGION=0.0 1.41 !Relative radii limiting trusted detector region

 DIRECTION_OF_DETECTOR_X-AXIS= 1.0 0.0 0.0
 DIRECTION_OF_DETECTOR_Y-AXIS= 0.0 1.0 0.0 ! 0.0 cos(2theta) sin(2theta)

!===================== JOB CONTROL PARAMETERS ===============================
 JOB= XYCORR INIT COLSPOT IDXREF DEFPIX INTEGRATE CORRECT


MAXIMUM_NUMBER_OF_JOBS=4  !Speeds-up COLSPOT & INTEGRATE on a Linux-cluster
MAXIMUM_NUMBER_OF_PROCESSORS=8!<32;ignored by single cpu version of xds
!SECONDS=0   !Maximum number of seconds to wait until data image must appear
!TEST=1     !Test flag. 1,2 additional diagnostics and images

!====================== GEOMETRICAL PARAMETERS ===============================
!ORGX and ORGY are often close to the image center, i.e. ORGX=NX/2, ORGY=NY/2
 ORGX= 555  ORGY= 624    !Detector origin (pixels).  ORGX=NX/2; ORGY=NY/2
```

```
 DETECTOR_DISTANCE= 45   !(mm)

 ROTATION_AXIS= 0.0 -1.0 0.0

! Optimal choice is 0.5*mosaicity (REFLECTING_RANGE_E.S.D.= mosaicity)
 OSCILLATION_RANGE=0.20            !degrees (>0)

 X-RAY_WAVELENGTH=1.2155          !Angstroem
 INCIDENT_BEAM_DIRECTION=0.0 0.0 1.0
 FRACTION_OF_POLARIZATION=0.99 !default=0.5 for unpolarized beam
 POLARIZATION_PLANE_NORMAL= 0.0 1.0 0.0

!====================== CRYSTAL PARAMETERS ===============================
 SPACE_GROUP_NUMBER=0
 UNIT_CELL_CONSTANTS= 41.6 79.1 83.8 90.1 103.0 89.7

! You may specify here the x,y,z components for the unit cell vectors if
! known from a previous run using the same crystal in the same orientation
!UNIT_CELL_A-AXIS=
!UNIT_CELL_B-AXIS=
!UNIT_CELL_C-AXIS=

!Optional reindexing transformation to apply on reflection indices
!REIDX=   0  0 -1  0  0 -1  0  0 -1  0  0  0

!FRIEDEL'S_LAW=FALSE !Default is TRUE.

!REFERENCE_DATA_SET= CK.HKL   !Name of a reference data set (optional)
!FIT_B-FACTOR_TO_REFERENCE_DATA_SET=TRUE ! default is FALSE

!=================== SELECTION OF DATA IMAGES ============================
!Generic file name and format (optional) of data images
! for Eiger, you should replace "master" with "??????" - see below:
 NAME_TEMPLATE_OF_DATA_FRAMES= ${datatemplate}_??????.h5
! this limits the number of frames to 999999
! Note: you can _not_ use "????" or "?????" but "00????" should also work
 LIB=/nfs/chess/sw/macchess/dectris-neggia-centos6.so

 DATA_RANGE=1 720
!EXCLUDE_DATA_RANGE= 53 58
!EXCLUDE_DATA_RANGE=  1  1
!EXCLUDE_DATA_RANGE= 888 905

 BACKGROUND_RANGE=6 10  !Numbers of first and last data image for background

 SPOT_RANGE=1 720       !First and last data image number for finding spots

!================= PARAMETERS CONTROLLING REFINEMENTS ======================
 REFINE(IDXREF)=BEAM AXIS ORIENTATION CELL  ! POSITION
 REFINE(INTEGRATE)= ORIENTATION POSITION BEAM ! CELL AXIS
 REFINE(CORRECT)=POSITION BEAM ORIENTATION CELL AXIS

!================== CRITERIA FOR ACCEPTING REFLECTIONS =====================
 VALUE_RANGE_FOR_TRUSTED_DETECTOR_PIXELS= 6000 30000 !Used by DEFPIX
                  !for excluding shaded parts of the detector.
```

```
 INCLUDE_RESOLUTION_RANGE=50 1.8 !Angstroem; used by DEFPIX,INTEGRATE,CORRECT

!WFAC1=1.0  !This controls the number of rejected MISFITS in CORRECT;
        !a larger value leads to fewer rejections.
!REJECT_ALIEN=20.0 ! Automatic rejection of very strong reflections

!============== INTEGRATION AND PEAK PROFILE PARAMETERS =====================
!Specification of the peak profile parameters below overrides the automatic
!determination from the images
!Suggested values are listed near the end of INTEGRATE.LP
!BEAM_DIVERGENCE=   0.80         !arctan(spot diameter/DETECTOR_DISTANCE)
!BEAM_DIVERGENCE_E.S.D.=   0.080 !half-width (Sigma) of BEAM_DIVERGENCE
!REFLECTING_RANGE=  0.780 !for crossing the Ewald sphere on shortest route
!REFLECTING_RANGE_E.S.D.=  0.113 !half-width (mosaicity) of REFLECTING_RANGE

! NUMBER_OF_PROFILE_GRID_POINTS_ALONG_ALPHA/BETA=21!used by: INTEGRATE
! NUMBER_OF_PROFILE_GRID_POINTS_ALONG_GAMMA=21     !used by: INTEGRATE

!DELPHI= 6.0!controls the number of reference profiles and scaling factors
!CUT=2.0    !defines the integration region for profile fitting
!MINPK=75.0 !minimum required percentage of observed reflection intensity

!======= PARAMETERS CONTROLLING CORRECTION FACTORS (used by: CORRECT) =======
!MINIMUM_I/SIGMA=3.0 !minimum intensity/sigma required for scaling reflections
!NBATCH=-1  !controls the number of correction factors along image numbers
!REFLECTIONS/CORRECTION_FACTOR=50   !minimum #reflections/correction needed
!PATCH_SHUTTER_PROBLEM=TRUE         !FALSE is default
!STRICT_ABSORPTION_CORRECTION=TRUE  !FALSE is default
!CORRECTIONS= DECAY MODULATION ABSORPTION

!===================== INDEXING PARAMETERS ================================
!Never forget to check this, since the default 0 0 0 is almost always correct!
!INDEX_ORIGIN= 0 0 0          ! used by "IDXREF" to add an index offset

!Additional parameters for fine tuning that rarely need to be changed
INDEX_ERROR=0.1 INDEX_MAGNITUDE=8 INDEX_QUALITY=0.8
 SEPMIN=4.0       ! default is 6 for other detectors
 CLUSTER_RADIUS=2 ! default is 3 for other detectors
!MAXIMUM_ERROR_OF_SPOT_POSITION=3.0
!MAXIMUM_ERROR_OF_SPINDLE_POSITION=2.0
!MINIMUM_FRACTION_OF_INDEXED_SPOTS=0.5


!=========== PARAMETERS DEFINING BACKGROUND AND PEAK PIXELS =================
!STRONG_PIXEL=3.0                              !used by: COLSPOT
!A 'strong' pixel to be included in a spot must exceed the background
!by more than the given multiple of standard deviations.

!MAXIMUM_NUMBER_OF_STRONG_PIXELS=1500000       !used by: COLSPOT

!SPOT_MAXIMUM-CENTROID=3.0                     !used by: COLSPOT

 MINIMUM_NUMBER_OF_PIXELS_IN_A_SPOT=3          !used by: COLSPOT
!This allows to suppress spurious isolated pixels from entering the
```

```
!spot list generated by "COLSPOT".

!NBX=3  NBY=3  !Define a rectangle of size (2*NBX+1)*(2*NBY+1)
!The variation of counts within the rectangle centered at each image pixel
!is used for distinguishing between background and spot pixels.

!BACKGROUND_PIXEL=6.0                          !used by: COLSPOT,INTEGRATE
!An image pixel does not belong to the background region if the local
!pixel variation exceeds the expected variation by the given number of
!standard deviations.

!SIGNAL_PIXEL=3.0                              !used by: INTEGRATE
!A pixel above the threshold contributes to the spot centroid

!FIXED_SCALE_FACTOR=TRUE  !Default is FALSE; used by : INIT,INTEGRATE

!============== DECISION CONSTANTS FOR FINDING CRYSTAL SYMMETRY =============
!Decision constants for detection of lattice symmetry (IDXREF, CORRECT)
! MAX_CELL_AXIS_ERROR=0.03 ! Maximum relative error in cell axes tolerated
! MAX_CELL_ANGLE_ERROR=2.0 ! Maximum cell angle error tolerated

!Decision constants for detection of space group symmetry (CORRECT).
!Resolution range for accepting reflections for space group determination in
!the CORRECT step. It should cover a sufficient number of strong reflections.
! TEST_RESOLUTION_RANGE=8.0 4.5
! MIN_RFL_Rmeas= 50 ! Minimum #reflections needed for calculation of Rmeas
! MAX_FAC_Rmeas=2.0 ! Sets an upper limit for acceptable Rmeas

eof
    cd ../.
done

for d in */ ; do
# ensure FRAMEMAX is the number of frames associated with that particular master file
# and FRAMEINC is the number of frames per well
    FRAME=1
    FRAMEMAX=24000
    FRAMEINC=15
    while [ $FRAME -lt $FRAMEMAX ]; do
        SECOND="$(echo "$FRAME + 14" | bc)"
        line="$(echo "${FRAME} ${SECOND}")"
        line_no_s="$(echo "${FRAME}_${SECOND}")"
        rm -rf ${d}/${line_no_s}
        mkdir ${d}/${line_no_s}
        cd $d
        cp XDS.INP ${line_no_s}/.
        cd $line_no_s
        sed -i "s/DATA_RANGE=.*/DATA_RANGE=${line}/g" XDS.INP
        sed -i "s/SPOT_RANGE=.*/SPOT_RANGE=${line}/g" XDS.INP
        sed -i "s/BACKGROUND_RANGE=.*/BACKGROUND_RANGE=${FRAME} ${SECOND}/g" XDS.INP
    echo "**************** $PWD ****************"
        xds_par # | tee output.log
    if [[ -e XDS_ASCII.HKL ]]; then
    # xdsstat spits out useful statistics (e.g. I/sigI for resolution shells) that
CORRECT.LP doesn't
```

```
        xdsstat > XDSSTAT.LP <<eof
        XDS_ASCII.HKL
eof

        cd ../../.
    else
        cd ../../.
        rm -rf ${d}/${line_no_s}
    fi
    FRAME="$(echo "$FRAME + $FRAMEINC" | bc)"
    done
done
```
